# Selective serotonin reuptake inhibitors (SSRIs) suppress Na^+^- dependent Mg^2+^ efflux in rat ventricular myocytes

**DOI:** 10.1016/j.jphyss.2025.100025

**Published:** 2025-05-12

**Authors:** Michiko Tashiro, Masato Konishi, Hana Inoue, Utako Yokoyama

**Affiliations:** Department of Physiology, Tokyo Medical University, Tokyo 160-8402, Japan

**Keywords:** Selective serotonin reuptake inhibitor (SSRI), Magnesium, Cardiomyocytes, Sertraline, Paroxetine, Fluvoxamine, Na^+^/Mg^2+^ exchange, Na^+^-dependent Mg^2+^ efflux

## Abstract

Na^+^/Mg^2+^ exchange transport, the Na^+^ gradient-driven Mg^2+^ extrusion system, plays a key role in cellular Mg^2+^ homeostasis. To date, the molecular entity and selective inhibitors of Na^+^/Mg^2+^ exchanger have not been fully explored. Intracellular free Mg^2+^ concentration ([Mg^2+^]_i_) was measured in ventricular myocytes acutely isolated from rat hearts. After soaking the cells in high-Mg^2+^ low-Na^+^ solution to increase [Mg^2+^]_i_, the addition of extracellular Na^+^ caused a decrease in [Mg^2+^]_i_. We analyzed the rate of decrease in [Mg^2+^]_i_ as Na^+^/Mg^2+^ exchange transport activity. The suppression of the rate of decrease in [Mg^2+^]_i_ caused by sertraline, a selective serotonin reuptake inhibitor (SSRI), was concentration dependent (IC_50_ 8.9 μM) and reversible. Other SSRIs, namely paroxetine and fluvoxamine, were less effective than sertraline. In conclusion, sertraline inhibited Na^+^/Mg^2+^ exchange transport more effectively than any previously reported inhibitors of Na^+^/Mg^2+^ exchanger. Sertraline could be used as a tool to characterize the functions of Na^+^/Mg^2+^ exchanger.

## Highlights

• Na^+^/Mg^2+^ exchange transport plays a key role in cellular Mg^2+^ homeostasis.
• We found that Na^+^/Mg^2+^ exchanger is inhibited by SSRIs, including sertraline.
• Sertraline is the most potent inhibitor of Na^+^/Mg^2+^ exchanger reported to date.
• Sertraline is a useful tool to characterize the functions of Na^+^/Mg^2+^ exchanger.

## 1 Introduction

Magnesium ions continue to inflow through the cell membrane via Mg^2+^ permeable channels driven by an electrochemical gradient, thus there must be active transport systems to maintain intracellular-free Mg^2+^ concentration ([Mg^2+^]_i_). The presence of the Na^+^/Mg^2+^ antiport system has been demonstrated in a variety of cell types, including hemocytes, hepatocytes, myocytes, neurons, and tubular epithelial cells in the kidney [1]. We have also characterized the Mg^2+^ extrusion system driven by Na^+^ concentration gradient across the cell membrane of cardiac ventricular myocytes [2], [3], [4], [5], [6]. However, the molecular entity of the Na^+^/Mg^2+^ exchanger responsible for cellular Mg^2+^ homeostasis remains controversial.

SLC41A1 and CNNM4 have been reported to be involved in Na^+^-dependent Mg^2+^ transport [7], [8]. Although SLC41A1 is ubiquitously expressed, no disruption of intracellular Mg^2+^ homeostasis has been observed in SLC41A1 knockout mice [9], suggesting that there are other mechanisms that maintain intracellular Mg^2+^. CNNM4 is localized on the basement membrane side of the distal renal tubules, which is important for the reabsorption of Mg ions to maintain the organismal Mg homeostasis. This is evidenced by the fact that CNNM4 KO animals and mutants develop hypomagnesemia [8]. While Mg^2+^ transport by CNNM4 has been reported to be Na^+^-dependent, it has been noted that its molecular size is too small for the exchanger [10]. Recently, CNNM4 have been suggested to regulate activities of Mg^2+^ permeable channels, such as TRPM7, which are involved in Mg^2+^ entry [11]. It is possible that the genetic knockout of a single molecule, SLC41A1 or CNNM4, is compensated for Mg^2+^ transport by other transporters, then the physiological phenomenon may not be observed. In this study, we used native cells to investigate the physiological characteristics of Mg^2+^ transport.

Several compounds have been reported to inhibit Na^+^/Mg^2+^ exchange transport, including imipramine (a type of tricyclic antidepressant, an inhibitor of monoamine neurotransmitter transporters, NET which are members of a Na^+^-dependent neurotranmitter transporter gene family), quinidine, and amiloride [12], [13], [14]. We previously reported that KB-R7943, a known inhibitor of the Na^+^/Ca^2+^ exchanger, inhibited Na^+^/Mg^2+^ exchanger with a half-maximal inhibitory concentration (IC_50_) of 21 μM at 25 ℃, which was more effective than imipramine (IC_50_: 59 μM) [15]. Since these inhibitors of Na^+^/Mg^2+^ exchange transport were originally recognized as inhibitors of Na^+^ symporters (e.g. imipramine for Na^+^/Cl^−^-dependent norepinephrine transporter) or Na^+^ antiporters (e.g. KB-R7943 for Na^+^/Ca^2+^ exchanger, amiloride for Na^+^/H^+^ exchanger)[16], it can be speculated that inhibitors of other Na^+^ symporters/antiporters might also inhibit Na^+^/Mg^2+^ exchange transport. In search of a more effective inhibitor, we found that sertraline, an SSRI (selective serotonin reuptake inhibitor) which inhibits a serotonin transporter (SERT) of the Na^+^/Cl^–^ - dependent transporter family, was a potent inhibitor of Na^+^/Mg^2+^ exchange activity with an IC_50_ even lower than that of KB-R7943.

## 2 Methods

### 2.1 Ethical approval

Ventricular myocytes were acutely isolated from the hearts of male Wistar rats (8–12 weeks old) under deep anesthesia using thiamylal sodium (150 mg/kg, IP). All experiments were performed under the procedures approved by the Institutional Animal Care and Use Committee (IACUC) of Tokyo Medical University (Permit Nos. R5–082).

### 2.2 Preparation

Rat ventricular myocytes were isolated enzymatically using the Langendorff perfusion system as previously described [6]. For enzymatic digestion, 0.5 mg/mL collagenase (type 2; Worthington, Lakewood, NJ), 0.05 mg/mL protease (type XIV; Sigma, St. Louis, MO) and 0.75 mg/mL BSA (Sigma) were used.

### 2.3 Solutions and chemicals

The basic perfusion solution was, the Ca^2+^-free Tyrode’s solution containing (mM) 135 NaCl, 5.4 KCl, 1.0 MgCl_2_, 0.33 NaH_2_PO_4_, 0.1 K_2_EGTA, 5.0 glucose, and 10 HEPES (pH 7.40 by NaOH). To raise intracellular Mg^2+^, the cells were soaked in Mg^2+^-loading solution composed of high-Mg^2+^ and low-Na^+^, containing (mM) 101 N-methyl-d-glucamine-Cl, 5.4 KCl, 18 MgCl_2_, 6 MgMs_2_, 0.33 NaH_2_PO_4_, 0.1 K_2_EGTA, 5.0 glucose and 10 HEPES (pH 7.40 by NaOH). To reduce intracellular Mg^2+^, the cells were soaked in Mg^2+^-depleting solution composed of high-K^+^ and low-Mg^+^, containing (mM) 140 KCl, 0.33 NaH_2_PO_4_, 0.1 K_2_EGTA, 5.0 glucose and 10 HEPES (pH 7.40 by KOH). High-K^+^ was employed to facilitate Mg^2+^ efflux by cell membrane depolarization.

**Fig. 1:**
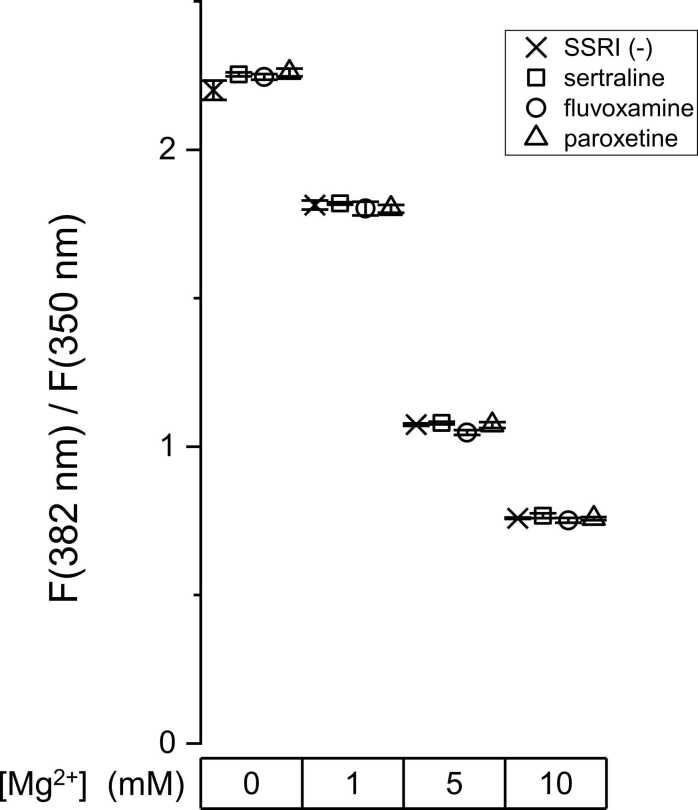
Selective serotonin reuptake inhibitors (SSRIs) did not change the Mg^2+^-related signal in vitro. The fluorescence signals [F(382)/F(350)] were measured from solutions including 50 μM furaptra with 50 μM sertraline (□), 50 μM fluvoxamine (○), 50 μM paroxetine (△), or without SSRI (×). Each symbol (mean ± SD, n = 3) was plotted as a function of [Mg^2+^] (mM) of solutions.

Sertraline-hydrochloride, paroxetine-hydrochloride-hemihydrate and fluvoxamine-maleate were obtained from Tokyo Chemical Industry (Tokyo, Japan). Furaptra AM (mag-fura-2 AM) was purchased from Invitrogen (Life Technologies, Carlsbad, CA). SSRIs were dissolved from their concentrated stock solutions in DMSO. The final drug concentration was less than 0.1 % during fluorescence measurements, which did not affect the [Mg^2+^]_i_ measurements (see Results, Fig. 1). All other chemicals were reagent grade.

### 2.4 Measurements of furaptra signals from single cells

Single ventricular myocytes were placed in a chamber on the stage of an inverted microscope (TE300; Nikon, Tokyo). Furaptra was loaded into the cells by incubation of the cells with 5 μM furaptra AM in normal Tyrode’s solution (containing 1 mM CaCl_2_) for 14 min at room temperature. The acetoxy methyl (AM) ester was washed out with the Ca^2+^-free Tyrode’s solution for at least 10 min. Furaptra fluorescence intensities were measured under Ca^2+^-free conditions at 25 °C to minimize possible cellular damage and interference with the furaptra fluorescence caused by Ca^2+^ overloading of the cells. The intracellular furaptra was excited with 350- and 382-nm light beams, and the fluorescence at 500 nm was detected from a single whole cell. The background fluorescence was measured for each cell at each excitation wavelength prior to furaptra loading to subtract from the total fluorescence measured as furaptra signals. The ratio of furaptra signals excited at 382 and 350 nm [R = F(382)/F(350)] was converted to [Mg^2+^]_i_ according to the equation:

[Mg^2+^]_i_ = K_D_ ∙ (R − R_min_) ∕ (R_max_ − R)·

The parameter values (K_D_ = 5.30 mM, R_max_ = 0.223, R_min_ = 0.969) were previously estimated in rat ventricular myocytes [17].

### 2.5 Analyses of Mg^2+^ efflux rates as the Na^+^/Mg^2+^ exchange transport activity

After the cells had been loaded with Mg^2+^ in Mg-loading solution for ∼ 2 h, superfusion with the Ca^2+^-free Tyrode's solution (i.e. the addition of Na^+^) induced a rapid decrease in [Mg^2+^]_i_. The initial rate of decrease in [Mg^2+^]_i_ was estimated by linear regression of data points spanning 180 s after the addition of extracellular Na^+^; this was considered to reflect the rate of Mg^2+^ efflux (Na^+^/Mg^2+^ exchange transport), as previously reported [5]. Based on the previous data, the rates are correlated with the initial [Mg^2+^]_i_ ([Mg^2+^]_i_ at time 0 calculated from linear regression) as in the following equation, relative Mg^2+^ efflux rate = V_max_ × X^N^ / (K^N^ + X^N^). We used these parameters (V_max_ = −2.52 μM/s, X = initial [Mg^2+^]_i_ – 0.78 mM, K = 0.73 mM, N = 0.913) to obtain relative Mg^2+^ efflux rate as the activity of the Na^+^/Mg^2+^ exchange transport.

### 2.6 Analyses of Mg^2+^ influx rates

To decrease [Mg^2+^]_i_ from the basal level (∼0.9 mM), the cells were incubated in Mg^2+^-depleting solution for 20 min at ∼30 °C. When the Mg^2+^-depleted cells were superfused with the Ca^2+^-free Tyrode's solution, [Mg^2+^]_i_ increased gradually to the basal level. We measured [Mg^2+^]_i_ at 4–5 min intervals for 60 min to calculate the Mg^2+^ influx rate as previously reported [18]. In brief, the time course of the recovery could be well fitted by a single exponential function of time (t), [Mg^2+^]_i_ (t) = A∙ exp(-t/τ)+ [Mg^2+^]_i_ (t = ꝏ), where A is a constant and τ is a time constant. Because the [Mg^2+^]_i_ recovery is caused by the influx of Mg^2+^, the first derivative of the recovery function is thought to reflect the rate of Mg^2+^ influx, $d{\left[Mg^{2+}\right]}_{i}\left(t\right)/\mathrm{dt}=\left(-A/\tau \right)\cdot \exp\left(-t/\tau \right)$.

We used the value of d[Mg^2+^]_i_ (t)/dt at time 0, −A / τ, as an index of the initial rate of Mg^2+^ influx.

### 2.7 Statistical analyses

Statistical values are presented as mean ± SD. Figures were generated with the program Origin (Ver. 9.6, Origin Lab, Northampton, MA, USA). Differences between groups were analyzed by the Mann-Whitney U-test with the significance level set at p < 0.05. (IBM SPSS Statistics 28).

## 3 Results

### 3.1 No interference of SSRIs with Mg indicators

The Mg^2+^-related fluorescence signal [F(382)/F(350)] was measured in quartz capillaries filled with solutions of various Mg^2+^ concentrations (0, 1, 5, or 10 mM) including 50 μM furaptra (potassium salt) and one SSRIs (50 μM sertraline, paroxetine or fluvoxamine). None of these compounds changed the fluorescence signal at 50 μM, as shown in Fig. 1.

### 3.2 The inhibitory effect of sertraline on Na^+^-dependent Mg^2+^ efflux

**Fig. 2:**
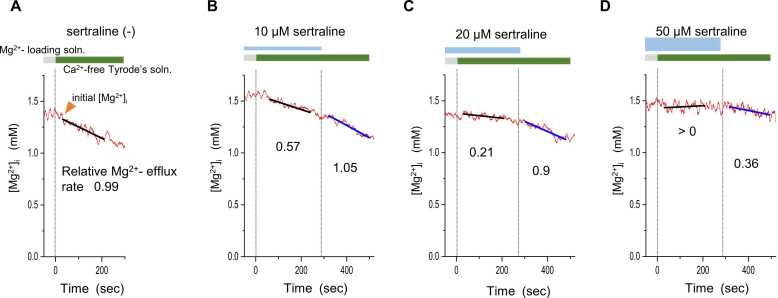
Representative records from separate experiments on the effects of sertraline. Mg^2+^-loaded cells were perfused with Ca^2+^-free Tyrode’s solution in the absence of sertraline (**A**), or in the presence of 10 μM (B), 20 μM (C) and 50 μM (D) sertraline as indicated at the top of each panel. [Mg^2+^]_i_ was measured continuously (0.2 s intervals) and smoothed with adjacent averaging of 51 data points (red lines). Black and blue lines were drawn by the least-squares fit to unsmoothed data points. Relative Mg^2+^-efflux rates (shown near the traces) were corrected by initial [Mg^2+^]_i_ (see Methods) and calculated from these slopes.

Switching the perfusate of a Mg^2+^-loaded cell from Na^+^-free (i.e., Mg^2+^-loading solution) to Na^+^-present (i.e., Ca^2+^-free Tyrode’s solution) decreased [Mg^2+^]_i_ as shown in Fig. 2. The decrease in [Mg^2+^]_i_ was slowed when sertraline was administered 10 min before Na^+^ addition, and the rate was partially recovered by removal of sertraline. The inhibitory effect was sertraline concentration dependent, with almost complete inhibition at 50 µM. At lower concentrations, recovery after sertraline washout was nearly complete.

**Fig. 3:**
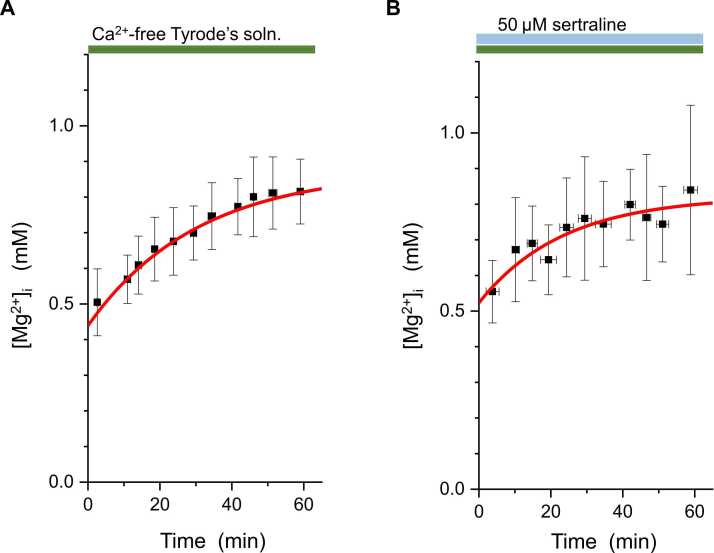
The effect of sertraline on Mg^2+^ influx. [Mg^2+^]_i_ of Mg^2+^-depleted cells (mean ± SD) was plotted as a function of time, in the Ca^2+^-free Tyrode’s solution in the absence (**A**, n = 5) or in the presence of 50 μM sertraline (**B**, n = 5). The red solid line is a single exponential function with mean values of the parameters obtained from each experiment (see Methods).

This suppression of the decrease in [Mg^2+^]_i_ was thought to be due to inhibition of Mg^2+^ efflux by sertraline, but it could also be due to an increase in Mg^2+^ influx. We therefore checked whether sertraline increased Mg^2+^ influx. As shown in Fig. 3, perfusing Mg^2+^ depleted cells with the Ca^2+^-free Tyrode’s solution caused an increase in [Mg^2+^]_i_ to the basal level. We analyzed the rate of [Mg^2+^]_i_ recovery as Mg^2+^ influx rate (see Methods for details). The average value of the rate was 0.27 ± 0.19 μM/s (n = 5, Fig. 3A) without sertraline, and it was not changed in cells treated with 50 µM sertraline (0.26 ± 0.19 μM/s, n = 5, Fig. 3B). These results suggest that sertraline does not increase Mg^2+^ influx but rather inhibits Mg^2+^ efflux.

### 3.3 Concentration dependence of the inhibitory effect of sertraline

**Fig. 4:**
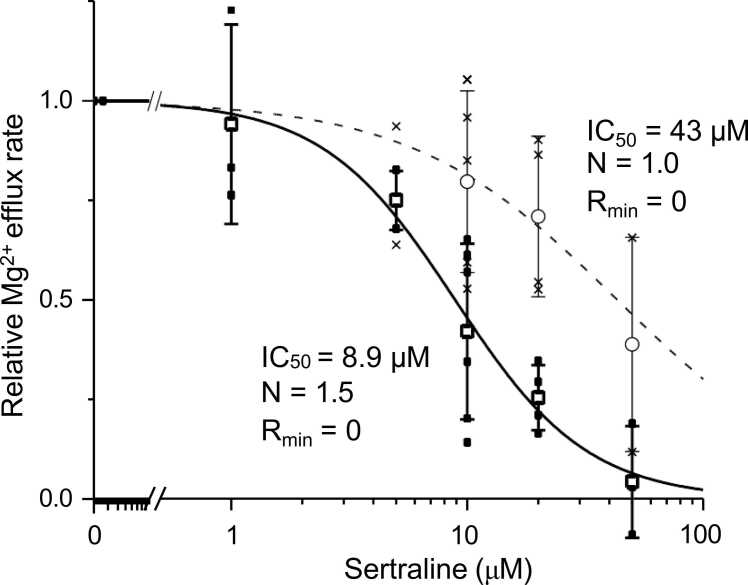
Sertraline concentration vs. Mg^2+^ efflux inhibition. Relative Mg^2+^ efflux rates obtained in each cell (■) and their mean ± SD values (□) were plotted as a function of sertraline concentration. The solid line indicates the least-squares fit of the data sets (■) by the Hill-type curve, [Relative Mg^2+^ efflux rate] = 1 − [X]^N^/([IC_50_]^N^ +[X]^N^), defined as the maximum value of 1 and the minimum value (R_min_) ≥ 0. [X] is sertraline concentration and N is the Hill coefficient. Relative Mg^2+^ efflux rates after washout of sertraline obtained in each cell (×) and their mean ± SD values (○) were also plotted. The dashed line was drawn by the least-squares fit of the data sets (×) in the same manner as described above for the solid line.

We repeated experiments similar to those shown in Fig. 2, from which we calculated the values relative to the initial [Mg^2+^]_i_ (i.e., relative Mg^2+^-efflux rates, see Methods). Fig. 4 shows the Hill-type fitted concentration-response curve (a solid line) with the relative Mg^2+^-efflux rates. The solid curve shows that sertraline inhibits Na^+^-dependent Mg^2+^ efflux (Na^+^/Mg^2+^ exchange transport) in a concentration-dependent manner with an IC_50_ value of 8.9 μM. Relative-Mg^2+^ efflux rates for 30–210 sec after sertraline washout (the slope of the blue line in Fig. 2) were also plotted and fitted as shown by the dashed line (IC_50_: 43 μM) in Fig. 4. Recovery after washout is also concentration dependent.

### 3.4 Other types of SSRIs also inhibited Mg^2+^ efflux

**Fig. 5:**
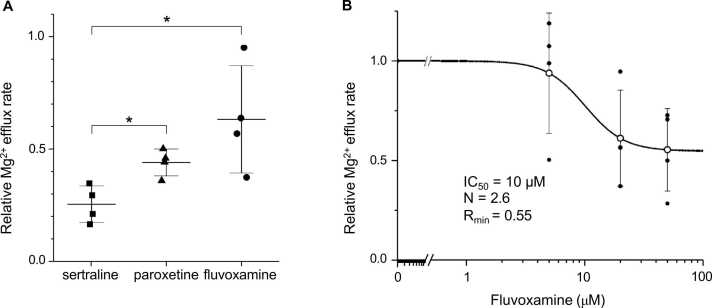
Comparison of the inhibitory effects of selective serotonin reuptake inhibitors (SSRIs). **A**, Relative Mg^2+^ efflux rates obtained in the presence of 20 μM of sertraline (■), paroxetine (▲), or fluvoxamine (●) were plotted together with mean ± SD values (bars). *p < 0.05 (Mann-Whitney U-test), **B,** Fluvoxamine concentration-response curve. Relative Mg^2+^ efflux rates obtained in each cell (•) and their mean ± SD values (○) were plotted against fluvoxamine concentration on the abscissa. The solid line indicates the Hill-type fitting curve analyzed in the same manner as described in Fig. 4 legend.

We examined the effects of two other types of SSRIs, paroxetine and fluvoxamine, which have chemical structures different from sertraline [19]. As shown in Fig. 5A, the inhibition of relative Mg^2+^ efflux rates by 20 µM sertraline was, on average, 75 ± 8 % (n = 4) compared to 56 ± 6 % (n = 4) for 20 µM paroxetine and 39 ± 24 % (n = 4) for 20 µM fluvoxamine. The inhibitory effect of the sertraline was significantly more potent than that of paroxetine (p = 0.021) or fluvoxamine (p = 0.029 by Mann-Whitney U-test) at 20 µM. Relative Mg^2+^ efflux rates obtained from experiments in the presence of 5, 20, or 50 μM fluvoxamine could be fitted with a concentration-dependent curve with an IC_50_ value of 10 μM (Fig. 5B). Note that maximal inhibition by fluvoxamine (45 %) was smaller than that by sertraline (100 %).

## 4 Discussion

### 4.1 Inhibitory effect of sertraline on Na^+^/Mg^2+^ exchange transport activity

We found that the selective serotonin reuptake inhibitor, sertraline, has an inhibitory effect on Na^+^/Mg^2+^ exchange transport activity. Sertraline is the most potent (IC_50_: ∼ 9 μM) inhibitor of the Na^+^/Mg^2+^ exchange transport reported to date. This inhibitory effect is not as potent as that on human serotonin transporter (SERT) (IC_50_: ∼ 0.4 μM) [20]. Sertraline is used in medicine as an antidepressant because of its SERT inhibitory effect. The mean C_max_ (maximum blood concentration) obtained from 6 healthy Japanese adult males at the maximum approved oral dose of 100 mg is 30.8 ng/mL (∼ 100 nM) [21]. Sertraline at does not appear to affect the Na^+^/Mg^2+^ exchanger at clinical doses, but it may be necessary to consider intracellular Mg^2+^ overload in patients with sertraline overdose, Mg^2+^ supplementation for antidepressant treatment, or abnormal metabolism of sertraline.

### 4.2 Effects of sertraline-induced changes in Na^+^ and K^+^ on Na^+^/Mg^2+^ exchanger

Lee et al. [22] reported that sertraline inhibits I_Na_ through Na^+^ channels (IC_50_: 6.1 μM) and suppressed K^+^ channels such as hERG, I_K1_, and I_Ks_ (IC_50_ values 0.7, 10.5, and 15.2 μM, respectively) expressed in HEK293 cells, using a whole-cell patch clamp technique. They also found that sertraline inhibits I_Ca_ with an IC_50_ of 2.6 μM in rat ventricular myocytes [22]. These IC_50_ values are also at the same level as that on the Na^+^/Mg^2+^ exchanger obtained in the present study. Simultaneous inhibition of these channels and/or transporters may disrupt the intracellular myocardial ion composition, resulting in physiological disruption, such as abnormal myocardial contraction and arrhythmia.

As sertraline thus alters intracellular ion dynamics, it is critical to consider whether the inhibition of Na^+^/Mg^2+^ exchange transport observed above is secondary to the alterations of other ion concentrations. A decrease in Na^+^ influx (by inhibiting the activity of Na^+^ channels and Na^+^- cotransporters, including SERT) should lead to activation (rather than the observed inhibition) of Na^+^/Mg^2+^ exchange transport by increasing the Na^+^ concentration gradient across the cell membrane as intracellular Na^+^ decreases [4]. We previously reported that Na^+^/Mg^2+^ exchange transport is little affected by K^+^ and Cl^−^ [3]. When sertraline inhibits K^+^ channels, cell membranes would be depolarized. We previously reported that the apparent rate of Mg^2+^ transport by Na^+^/Mg^2+^ exchange depends slightly on the membrane potential: facilitation is by depolarization and inhibition is by hyperpolarization with no sign of reversal between −120 and 0 mV [23]. Since Mg^2+^ efflux by Na^+^/Mg^2+^ exchange transport is facilitated by depolarization, it is unlikely that the inhibition of Mg^2+^ efflux is due to modulation of K^+^ channels by sertraline. The effect of intracellular Ca^2+^ should be minimal, because the experiments in the present study were performed under Ca^2+^-free conditions. It is therefore unlikely that sertraline-induced changes in other ion dynamics are responsible for the inhibition of Na^+^/Mg^2+^ exchange transport.

### 4.3 The comparison of the inhibitory potency of sertraline and fluvoxamine in SERT and the Na^+^/Mg^2+^ exchanger

Two other SSRIs, paroxetine and fluvoxamine, also inhibited Na^+^/Mg^2+^ exchange transport. Sertraline was the most effective among these 3 drugs in comparison at a concentration of 20 μM (see Fig. 5A). Regarding the potency of sertraline and fluvoxamine, concentration-response curves (see Fig. 4 and Fig. 5B) show only a slight difference in IC_50_ between these drugs, but the maximum effect of fluvoxamine was about half that of sertraline.

Mortensen et al. reported that the IC_50_ of sertraline for human SERT was twice that of fluvoxamine, and more than 10 fold that of paroxetine [20]; as for the inhibitory effect on SERT, sertraline is less potent than paroxetine and fluvoxamine. Interestingly, sertraline is a more potent inhibitor of the Na^+^/Mg^2+^ exchange transport than fluvoxamine. Structural analysis showed that these SSRIs regulate SERT activity by occupying its central substrate binding site [19]. It is therefore of interest for future studies to compare to distinguish the different modes of action of SSRIs between SERT and Na^+^/Mg^2+^ exchanger.

In conclusion, this study revealed the most potent inhibitor of the Na^+^/Mg^2+^ exchanger of those reported. Sertraline could be used as a tool to characterize the functions of the Na^+^/Mg^2+^ exchanger.

## CRediT authorship contribution statement

**Tashiro Michiko:** Writing – original draft, Visualization, Validation, Project administration, Methodology, Investigation, Funding acquisition, Formal analysis, Data curation, Conceptualization. **Konishi Masato:** Writing – review & editing, Validation, Supervision, Formal analysis, Conceptualization. **Inoue Hana:** Writing – review & editing, Validation, Supervision, Project administration, Conceptualization. **Yokoyama Utako:** Writing – review & editing, Validation, Supervision, Project administration, Conceptualization.

## Consent for publication

All the authors have approved the publication of the manuscript.

## Ethics approval and consent to participate

All experiments were performed under the procedures approved by the Institutional Animal Care and Use Committee (IACUC) of Tokyo Medical University (Permit Nos. R5–082).

## Funding

This work was supported by JSPS KAKENHI [grant numbers JP20K11518, JP24K08754].

## Conflict of interest

The authors declare no competing interests.

## Data Availability

Data will be made available on reasonable request.

## References

[1] Günther T. (2007). Na+/Mg2+ antiport in non-erythrocyte vertebrate cells. Magnes Res.

[2] Tashiro M., Inoue H., Konishi M. (2009). Metabolic inhibition strongly inhibits Na+-dependent Mg2+ efflux in rat ventricular myocytes. Biophys J.

[3] Tashiro M., Tursun P., Miyazaki T., Watanabe M., Konishi M. (2006). Effects of intracellular and extracellular concentrations of Ca2+, K+, and Cl- on the Na+-dependent Mg2+ efflux in rat ventricular myocytes. Biophys J.

[4] Tashiro M., Tursun P., Konishi M. (2005). Intracellular and extracellular concentrations of Na+ modulate Mg2+ transport in rat ventricular myocytes. Biophys J.

[5] Tursun P., Tashiro M., Konishi M. (2005). Modulation of Mg2+ efflux from rat ventricular myocytes studied with the fluorescent indicator furaptra. Biophys J.

[6] Tashiro M., Konishi M. (2000). Sodium gradient-dependent transport of magnesium in rat ventricular myocytes. Am J Physiol Cell Physiol.

[7] Kolisek M., Launay P., Beck A., Sponder G., Serafini N., Brenkus M. (2008). SLC41A1 is a novel mammalian Mg2+ carrier. J Biol Chem.

[8] Yamazaki D., Funato Y., Miura J., Sato S., Toyosawa S., Furutani K. (2013). Basolateral Mg2+ extrusion via CNNM4 mediates transcellular Mg2+ transport across epithelia: a mouse model. PLoS Genet.

[9] Ilenwabor B.P., Franken G.A.C., Sponder G., Bos C., Racay P., Kolisek M. (2022). SLC41A1 knockout mice display normal magnesium homeostasis. Am J Physiol Ren Physiol.

[10] Arjona F.J., de Baaij J.H.F. (2018). CrossTalk opposing view: CNNM proteins are not Na(+) /Mg(2+) exchangers but Mg(2+) transport regulators playing a central role in transepithelial Mg(2+) (re)absorption. J Physiol.

[11] Bai Z., Feng J., Franken G.A.C., Al'Saadi N., Cai N., Yu A.S. (2021). CNNM proteins selectively bind to the TRPM7 channel to stimulate divalent cation entry into cells. PLoS Biol.

[12] Nakayama S., Nomura H. (1995). Mechanisms of intracellular Mg2+ regulation affected by amiloride and ouabain in the guinea-pig taenia caeci. J Physiol.

[13] Flatman P.W., Smith L.M. (1990). Magnesium transport in ferret red cells. J Physiol.

[14] Gunther T., Vormann J. (1995). Reversibility of Na+/Mg2+ antiport in rat erythrocytes. Biochim Biophys Acta.

[15] Tashiro M., Inoue H., Konishi M. (2010). KB-R7943 inhibits Na+-dependent Mg2+ efflux in rat ventricular myocytes. J Physiol Sci.

[16] Frelin C., Barbry P., Vigne P., Chassande O., Cragoe E.J., Lazdunski M. (1988). Amiloride and its analogs as tools to inhibit Na+ transport via the Na+ channel, the Na+/H+ antiport and the Na+/Ca2+ exchanger. Biochimie.

[17] Watanabe M., Konishi M. (2001). Intracellular calibration of the fluorescent Mg2+ indicator furaptra in rat ventricular myocytes. Pflug Arch.

[18] Tashiro M., Inoue H., Konishi M. (2014). Physiological pathway of magnesium influx in rat ventricular myocytes. Biophys J.

[19] Coleman J.A., Gouaux E. (2018). Structural basis for recognition of diverse antidepressants by the human serotonin transporter. Nat Struct Mol Biol.

[20] Mortensen O.V., Kristensen A.S., Wiborg O. (2001). Species-scanning mutagenesis of the serotonin transporter reveals residues essential in selective, high-affinity recognition of antidepressants. J Neurochem.

[21] Kamijima K., Otsubo T., Ota A., S. N, Inamoto J.H.T., Fujiwara A. (1997). Phase Ⅰ study of sertraline hydrochloride (CP-51, 974-1) -Single dose study-. Jpn J Neuropsychopharmacol.

[22] Lee H.A., Kim K.S., Hyun S.A., Park S.G., Kim S.J. (2012). Wide spectrum of inhibitory effects of sertraline on cardiac ion channels. Korean J Physiol Pharmacol.

[23] Tashiro M., Tursun P., Miyazaki T., Watanabe M., Konishi M. (2002). Effects of membrane potential on Na+ -dependent Mg2+ extrusion from rat ventricular myocytes. Jpn J Physiol.

